# California Cannabis Research Briefing, April 2023: Meeting Summary

**DOI:** 10.1089/can.2024.0074

**Published:** 2024-12-02

**Authors:** Margarete C. Kulik, Youn Ok Lee, Van Butsic, Timmen L. Cermak, Ziva D. Cooper, Dominic Corva, Thomas D. Marcotte, Dilara K. Üsküp, Tracy Richmond McKnight, Agnes Balla

**Affiliations:** ^1^ Tobacco-Related Disease Research Program, Research Grants Programs Office, University of California Office of the President, Oakland, California, USA.; ^2^ Department of Environmental Science, Policy, & Management, University of California, Berkeley, Berkeley, California, USA.; ^3^ California Society of Addiction Medicine, San Francisco, California, USA.; ^4^ Private Practice, General & Addiction Psychiatry, San Francisco, California, USA.; ^5^ Semel Institute for Neuroscience and Human Behavior, Department of Psychiatry and Behavioral Sciences, University of California, Los Angeles, California, USA.; ^6^ UCLA Center for Cannabis and Cannabinoids, University of California, Los Angeles, California, USA.; ^7^ Humboldt Institute for Interdisciplinary Marijuana Research, Cal Poly Humboldt, Arcata, California, USA.; ^8^ Department of Sociology, Cannabis Studies Program Lead, Cal Poly Humboldt, Arcata, California, USA.; ^9^ Center for Medicinal Cannabis Research, University of California San Diego, San Diego, California, USA.; ^10^ Department of Psychiatry, University of California San Diego, San Diego, California, USA.; ^11^ Department of Internal Medicine, Charles R. Drew University, Los Angeles, California, USA.; ^12^ Department of Family Medicine, Center for HIV Identification, Prevention, and Treatment Services, University of California, Los Angeles (UCLA), Los Angeles, California, USA.; ^13^ Research Policy Analysis and Coordination, University of California Office of the President, Oakland, California, USA.

**Keywords:** cannabis, THC, policy, meeting, University of California

## Abstract

On April 28, 2023, the University of California Office of the President, in partnership with the California Department of Cannabis Control (DCC), hosted the California Cannabis Research Briefing. The California Cannabis Research Briefing brought together researchers and state agencies/policymakers to discuss pertinent policy issues on cannabis within the state. Researchers across six different topic areas (environment, cannabis markets, social equity matters, public health, medicinal cannabis use, and public safety) provided brief explanations of their research and its policy implications. A moderated discussion with stakeholders followed these presentations. The goals of this event were to highlight research that can inform policy issues relevant to the state, and to discuss how research can be incorporated into the cannabis policy landscape.

## California Cannabis Research Briefing, April 2023: Meeting Summary

On April 28, 2023, the University of California Office of the President, in partnership with the California Department of Cannabis Control (DCC), hosted the California Cannabis Research Briefing. The California Cannabis Research Briefing brought together researchers and state agencies/policymakers to discuss pertinent policy issues on cannabis within the state. Researchers across six different topic areas (environment, cannabis markets, social equity matters, public health, medicinal cannabis use, and public safety) provided brief explanations of their research and its policy implications. A moderated discussion with stakeholders followed these presentations. The goals of this event were to:

- Highlight research that can inform policy issues relevant to the State.
- Discuss how research can be incorporated into the cannabis policy landscape.

Key issues and policy recommendations discussed during the event are listed below.

## Cannabis and the Environment

The Cannabis and the Environment panel (Table 1) consisted of Van Butsic (UC Berkeley), Patricia Holden (UC Santa Barbara), Christopher Dillis (UC Berkeley), Houston Wilson (UC Riverside), and Evan Mills (Lawrence Berkeley National Laboratory). The group discussed how cannabis policy intersects with environmental issues.

**Table 1.** Cannabis and the Environment Panel

**Presentation**	**Key policy issues and recommendations**
Overview of environmental issues in cannabis cultivation, *Van Butsic, PhD* (Keynote and session moderator)	Issue: There are critical knowledge gaps in how, how much, and where cannabis is grown in California, and differential impacts of cultivation techniques need to be further studied. Recommendation: Policy changes that make it easier for researchers to interact with plant material and run field trials could help in filling knowledge gaps.
Surface Water Emissions from Cannabis Cultivation Sites: Quantity, Quality, Toxicity, and Relationships to Farmers' Practices, *Patricia Holden, PhD*	Issue: Dr. Holden described her multipronged approach to sampling surface water emissions and measuring plant product residues to understand pesticide use and toxicity among legal cannabis growers in Santa Barbara County. Learning how to conduct this type of work given the different layers of regulations from local to state to the federal level is a challenge. Recommendations: Assistance from the state on the following would benefit researchers navigating conducting work: (1) Understanding local and state-level policies and regulations to better contextualize production. (2) Understanding differences across cultivation formats and how to inform consumers about the impacts on products they consume.
The Threat of Wildfire to the Licensed California Cannabis Industry, *Christopher Dillis, PhD*	Issue: Dr. Dillis provided an overview of his work on surveying licensed cannabis farmers on the impacts of wildfires and mitigation strategies for production loss. On this survey, 70% of survey respondents reported being given evacuation notices while almost 50% reported losing site access. The ability for farmers to maintain cannabis site access during wildfires to perform mitigative interventions is likely the most effective solution to minimize production impacts. Recommendations: (1) The state should support and facilitate Agricultural Pass Programs for cannabis farmers, which allow farmers to access their sites during mandatory evacuations. Agricultural Pass Programs are typically available to most other type of farmers. (2) The state should add a mechanism to record wildfire-related crop loss to current annual reporting protocols. Sophisticated estimation of potential production losses, and plans for mitigating losses, will require data with greater coverage and spatial resolution.
Development of Best Management Practices for California Cannabis Production, *Houston Wilson, PhD*	Issue: Dr. Wilson explained his work around understanding current cannabis production practices and the limitations in conducting field work. Recommendation: Policy changes should support researchers' access to cannabis farms and the ability to conduct field trials.
Environmental Justice: The Disproportionate Environmental Impacts of Indoor Cannabis Cultivation on Marginalized and Lower-Income Populations, *Evan Mills, PhD*	Issue: Dr. Mills detailed how indoor cannabis cultivation is highly energy-intensive, which disproportionately inflicts environmental harms on disadvantaged workers and surrounding communities. Recommendation: Policy makers need to intervene and consider the following tiered approaches to veer indoor cannabis cultivation to be environmentally sustainable (listed from most ambitious to least): (1) Prohibit indoor cultivation, particularly in urban areas. (2) Promote sustainable outdoor cultivation. (3) Require 100% on-site renewables. (4) Improve buffers where people gather (e.g., around homes, schools). (5) Enhance transparency. (6) Curb policies favoring indoor cultivation. (7) Prohibit colocation of fossil-fuel power plants at grows. (8) Measure pollutants. (9) Improve enforcement (unburden citizen groups). (10) Review social equity programs that inadvertently incentivize environmental inequities.

## California Cannabis Markets

The California Cannabis Markets panel (Table 2) addressed issues affecting the regulated cannabis market, including barriers to entry into the legal market, market trends, the feasibility of potency taxes, issues faced by legacy growers, and the effects of local county cannabis cultivation bans. The panel consisted of Dominic Corva (Cal Poly Humboldt), Olenna Sambucci (UC Davis), Brad Rowe (UCLA), Keith Taylor (UC Davis), Margiana Peterson-Rockney (UC Berkeley), and Michael Polson (UC Berkeley).

**Table 2.** California Cannabis Markets Panel

**Presentation**	**Key policy issues and recommendations**
The Illicit Market and Barriers to Entry, *Dominic Corva, PhD (Keynote and session moderator)*	Dr. Corva discussed barriers and challenges to entry into the legal cannabis market from the perspective of small and medium cannabis enterprises trying to join and remain part of the legal market. He described the issue of stacking, a term for growers with multiple permits for a single piece of property, comparing Humboldt County and Santa Barbara County.
Legal Cannabis Prices and the Economic Impacts of Regulations, *Olena Sambucci and Robin Goldstein, PhD*	Dr. Sambucci reported on trends in retail pricing and THC level, showing that retail prices declined while THC percentage in cannabis increased. In addition, consumer demand for high THC flower is shaping supply chain tendencies toward increased potency, since those products have a higher profit margin.
Non-Compliant and Unlicensed Operations Pushing Industry to the Edge: Emergent Findings from Potency Tax Feasibility and Cannabis Sociodemographic Assessments, *Brad Rowe, MPP*	Issue: Mr. Brad Rowe reported on the feasibility of adding potency taxes, finding that while supported by policymakers to stabilize tax revenues, to address potency inflation, and to address public health concerns, the distressed industry may oppose the shift, unless the potency tax was offset by phasing out other taxes. Recommendations: Mr. Brad Rowe emphasized the need to disincentivize laboratory shopping and to help the industry stabilize from unlicensed competition, market freefall, and escalating operational expenses; special emphasis on assistance to social equity licensees. He also recommended strengthening and promoting the evidence basis for a potency tax as a public health tool.
Small vs. Large Cannabis Entities: Collective Action Opportunities and Challenges, *Keith Taylor, PhD*	Issue: Dr. Taylor described his work assessing whether local jurisdictions administering Proposition 64 have achieved the goals of Proposition 64, particularly in encouraging positive local and state economic outcomes and fairness in the market for legacy growers. A challenge for California is that Proposition 64 places a substantial regulatory and enforcement responsibility on local government, while at the same time localities are underresourced. Recommendations: (1) Treat cannabis similar to any other agricultural commodity. (2) Put legacy cannabis businesses on parity with other enterprises. (3) Have guidance for local jurisdictions, particularly those that are resource strapped, on forming joint-powers-authority. (4) Enhance opportunities for cannabis cooperatives.
Local regulations and effects of cultivation bans on cannabis markets, *Margiana Petersen-Rockney, PhD, and Michael Polson, PhD*	Issue: Dr. Petersen-Rockney and Dr. Polson explained their work in examining causes and effects of local cannabis cultivation bans. Their preliminary findings show that: (1) Local cultivation bans range from softer (e.g., warnings, abatement notices) to stricter approaches (e.g., aggressive enforcement actions to proactively eliminate cultivations like raid). (2) Stricter enforcement has doubtful long-term success and significant short-term consequences. (3) Softer approaches, led by code enforcement, can ameliorate negative outcomes by fostering norms among cannabis cultivators. (4) Bans require significant public resources and can divert resources from existing enforcement and policy priorities. (5) Bans can focus on eradication not remediation. (6) Bans limit tools to regulate and control cultivation. Bans do not necessarily stop cultivation, but instead eliminate the ability to regulate for social and environmental concerns. (7) Bans raise concerns over social equity. (8) Bans can extend to personal and medical cultivation and access. Recommendations: (1) Smaller scale exceptions to local bans administered at the state level. (2) Reform local enforcement approaches, making unlicensed cultivation a civil, not criminal, matter. (3) Redirect the way state agencies assist counties that have banned cultivation. (4) Create a state oversight commission to review local-level ban enforcement to ensure fairness, personal access, and reduce legal issues.

## Social Equity

The Social Equity panel (Table 3) consisted of Dilara Üsküp (UCLA & Charles R. Drew University of Medicine and Science), Robert Chlala (UCLA & CSU Long Beach), Stella Beckman (UC Davis), and Laura Herrera (UC Berkeley). This panel explored the impact of California social equity licensing programs, especially those impacting operators, workers, and surrounding communities, and its impacts on marginalized communities and those impacted by the War on Drugs.

**Table 3.** Social Equity Panel

**Presentation**	**Key policy issues and recommendations**
Equity and Equality in the California Cannabis Industry, *Dilara Üsküp, PhD, PhD (Keynote and session moderator)*	Issue: Dr. Üsküp described challenges to the cannabis industry in California, including continued federal prohibition, unfair business practices by larger industry players, and the high costs of business operations in the state. She underscored how these factors exacerbate inequity and inequality, among applicants and licensees, in social equity programs. Recommendations: Dr. Üsküp highlighted the importance of establishing regulations that foster fairness and equity in the California cannabis market. She recommended the following strategies: (1) Administering incentives for joining the legal market. (2) Implementing progressive enforcement of the unlicensed market. (3) Establishing add-on licenses (at a reduced cost) for operators to transport their own product(s) to other cannabis businesses (e.g., cannabis retailers), thereby reducing their cost of goods sold. (4) Setting limits for municipalities on license processing and occupancy permitting time lines. (5) Improving state oversight of local municipalities and social equity programs to aid trust building and accountability. (6) Conditioning municipal grants on standardized transparency and accountability measures. (7) Providing opportunities for early-stage and continued stakeholder feedback, and timely access to regulatory support. (8) Financial support for greater industry-wide technology infrastructure, particularly tools to process state and local applications renewals, and tax remittances. (9) Providing licensees with access to aggregated track and trace data and basic analysis illustrating product, geographic, quantity, and price trends. (10) Alignment of track and trace API language with cannabis business POS systems and industry standards. (11) Creating a state liaison and template for aligning California Environmental Quality Act (CEQA) and California Department of Fish and Wildlife requirements. Allowing municipalities to use these standards as a baseline to allow their local cannabis businesses *de facto* approval as long as they are in good standing with the city or county. (12) Addressing process duplication, conflicting regulations, and other challenges and inefficiencies inherent to state and local dual licensing requirements. (13) Implementing “farm-to-fork” model and tax incentives to support access for medical users. (14) Significant and ongoing support for black- and brown-owned or operated businesses. Exploration of noncannabis tax revenue resources to aid in the remediation of the historic harms from the War on Drugs.
Essential Perspectives on Equity: Understanding Cannabis Worker Experiences of a Changing Industry, *Robert Chlala, PhD*	Issue: Dr. Chlala discussed how cannabis workers experience a wide range of conditions dependent on numerous factors: local policy (including equity), company size, unionization, industry sector, and race/gender/disability. Cannabis workers' motivations are often grounded in serving patients/consumers, plants, and the community—a key resource that can help realize certain regulatory and social goals and improve impact. Recommendations: (1) Research and data should be developed as a collaborative process among researchers/policymakers and cannabis workers to implement regulations and develop future equity programs. (2) Workers are benefiting from having a voice via unions and need a voice in policymaking, including in unregulated spaces. Policymakers must monitor the rise in fake unions and support via a community-based model the implementation of existing workforce protections, including training and labor peace agreements. (3) Investing in training is a key priority for workers, especially for deepening medical and scientific knowledge. Workforce development should not assume a lack of expertise but value the important knowledge that helped create the industry's value. (4) Collecting state/local wage, hour, benefits data from licensed operators and through worker data will help better develop profiles of economic impact—which holds much promise. (5) Consolidation is appearing as a key trend worsening conditions and should be monitored closely, including to understand how the integration into larger industrial agricultural and distribution processes is worsening workforce experiences and economic mobility. (6) Worker-owned cooperatives are a key potential path to meet equity ownership and workforce goals and improve the overall conditions that workers face. (7) The diversity of worker experiences is an asset—it is radically underestimated and in fact is part of what makes cannabis an opportunity to develop model economic and public health practices.
Cannabis Farmworkers' Safety and Health, *Stella Beckman, PhD, MPH*	Issue: Dr. Stella Beckman detailed that workplace hazards and exposures experienced by cannabis workers are similar to industrial farms (e.g., dust, chemicals, and ergonomic hazards). Safety factors unique to the cannabis industry included geographic isolation and criminalization of the industry. Recommendations: (1) Easing financial and administrative barriers to licensing will provide access for labor organizing as well as enabling employers to access worker safety resources such as Cal/OSHA's consultation program. (2) Education and training programs based on social media campaigns and community outreach in cannabis-growing regions will be valuable methods for reaching cannabis workers in locations that are inaccessible to regulators or when employers are uncooperative.
Social Impacts of Cannabis Equity Programs, *Laura Herrera, MPA*	Issue: Ms. Laura Herrera described that while DCC's initial equity program and local social equity programs provided modest and necessary support, policymakers must substantially increase and stabilize funding to maintain the positive momentum of current programs and help scale up to overcome the hurdles equity operators face. Recommendations: (1) Allocate a portion of state social equity tax revenues to be recycled to sustain grants to social equity operators. (2) Standardize criteria for equity verification to meet intentions of Prop 64/SB1294. (3) Encourage and facilitate cross-jurisdictional pooling of resources for permit counties by providing guidance for local governments to form joint-powers-authorities that would centralize professional staff technical assistance and standardize and coordinate regulatory policies and practices. (4) Facilitate dialogue and open communication among equity programs and their participants to share best practices and react quickly to market and community feedback.

API, application programming interface; DCC, Department of Cannabis Control; POS, point of sale.

## Cannabis and Public Health

The Cannabis and Public Health panel (Table 4), moderated by Dr. Timmen Cermak, discussed a wide range of public health issues raised by cannabis and its legal regulation. Dr. Timmen Cermak was joined by Daniele Piomelli (UC Irvine), Lynn Silver (Public Health Institute), Pamela Ling (UCSF), and Mariaelena Gonzalez (UC Merced).

**Table 4.** Cannabis and Public Health Panel

**Presentation**	**Key policy issues and recommendations**
The Role of Public Health in Cannabis Legalization, *Timmen Cermak, MD (Keynote and session moderator)*	Issue: Dr. Cermak emphasized the paramount interest in protecting the public's health that is explicitly embedded in legislation legalizing cannabis, basic science research establishing the addictive nature of cannabis, potential harms from consuming high concentrations of THC (including increased rates of psychosis), and the responsibility legislators and regulators have for understanding the products being legalized for public consumption.
Impact of early-life cannabis exposure: lesson from basic science (*Daniele Piomelli, PhD*)	Issue: Dr. Piomelli discussed recent laboratory research findings suggesting cannabis exposure during adolescence produces evidence of impaired metabolic, physiologic, and immunologic function during later early adult life. These changes may have unpredictable consequences on both physical and mental health. Recommendations: Additional research is critical in understanding the risks of cannabis exposure. The state should create a California research agenda and prioritize funding the following types of studies and research areas: Longitudinal population studies Ecologically relevant studies in animal models Ecologically relevant studies in the human laboratory setting Persistent impact of cannabis on adolescents Impact of prenatal and neonatal cannabis exposure Impact of high-potency cannabis
Cannabis use: some issues regarding health equity (*Mariaelena Gonzalez, PhD*)	Issue: Dr. Gonzalez presented research on the impact of cannabis legalization and health equity, with an emphasis on university student populations, pregnant women, and immigrant communities. Among university students, recent research estimated that ∼3 in 10 people who use cannabis have cannabis- use disorder. For people who begin using cannabis before age 18, the risk of developing cannabis-use disorder is even greater. Among pregnant women, there is increasing evidence that use of cannabis is associated with adverse effects for fetal, neonatal, and neurodevelopmental outcomes when cannabis is used during pregnancy, yet use of cannabis during pregnancy increased after legalization. Among immigrants, if an immigrant tells an immigration officer that they have used cannabis, sold it, or even possessed it (even in states where it is legal), they can be denied entry in the United States, deemed inadmissible, have their citizenship applications denied, and can ultimately be deported. Recommendations: Dr. Gonzalez's recommendations for state action to improve the health of university student populations included: (1) Harmonizing the age limit for medical and recreational cannabis use. (2) Decreasing the attractiveness of cannabis products to adolescents, including by having plain packaging, graphic warning labels, and banning flavored products. Recommendations for state action to improve the health of pregnant women included: (3) Funding education campaigns on the risks of using cannabis among pregnant and breastfeeding mothers. (4) Funding studies on basic science, the impact of high THC strains on fetal development and decision-making regarding use of cannabinoids during pregnancy. Recommendations for state action to improve the health equity of immigrants included: (5) Putting pressure on the federal government to change scheduling and pardon those previously deported. (6) Ensuring strong medical privacy laws in California to protect medicinal cannabis users. (7) Considering the impacts of cannabis regulation on immigrants.
How can policy research contribute to regulatory protection of public health, children and youth (*Lynn Silver, MD, MPH, FAAP*)	Issue: Dr. Lynn Silver discussed how cannabis retail density affects cannabis use during pregnancy, the effectiveness of current California warning labels, and accelerating the translation of research into cannabis policies. Recommendations: (1) Given that the number and location of retail licensees are associated with greater odds of cannabis use during pregnancy, which is recognized as harmful, the state should consider limiting the number of licenses in residential areas to help constrain increases in consumption during pregnancy. (2) Current warning information is incomplete and ineffectively communicated. Therefore, the state should consider the best evidence on effective communication approaches to inform health information and required warnings and continue to adapt as further evidence emerges. (3) To ensure that the translation of public health research is reflected in cannabis policymaking, regulators should consider creating a standing medical and public health scientific advisory body without conflicts, strengthen public health and product regulation, internal expertise, and maintain funding of ongoing policy research beyond the requirement in Proposition 64.
Second-hand smoke, smoke-free laws, and the intersection with tobacco (*Pamela Ling, MD, MPH*)	Issue: Dr. Ling presented research from tobacco cessation efforts, including second-hand smoke, smoke-free laws, and the intersection of cannabis use with tobacco. Recommendations: (1) Dispensaries should not allow on-site vaping or smoking. (2) There should be no exceptions to clean air policies for cannabis. (3) Do not reintroduce smoking into smoke-free restaurants and bars. (4) Independent research without industry conflict of interest is important.

## Medicinal Cannabis Benefits and Risks

This panel provided a background on rates of medicinal cannabis use both nationally and within one of the University of California's health care systems (UCLA), while also discussing known therapeutic effects of cannabis and cannabinoids, hazards associated with use, the need for research to further understand both potential harms and benefits of medical cannabis use, and priority areas that need to be addressed to shape public policy that is in the interest of public health (Table 5). The panel members included Ziva Cooper (UCLA), Lillian Gelbert (UCLA), Gregory Marcus (UCSF), and Igor Grant (UCSD).

**Table 5.** Medicinal Cannabis Benefits and Risks Panel

**Presentation**	**Key policy issues and recommendations**
Known benefits of cannabis use and risk factors for cannabis misuse, *Ziva Cooper, PhD (Keynote and session moderator)*	Issue: Dr. Ziva Cooper described the complexities of considering and researching the therapeutic effects of cannabis. Cannabis is a plant with hundreds of constituents, many of which are known (or hypothesized) to be biologically active, all present in different concentrations across plants and cannabis products, which are administered across a range of modalities. As such, discussing the medical and adverse effects of cannabis requires precision with respect to its chemical composition and product type. Consequences of cannabis use will also depend on motives for use and the individual as differences in outcomes can exist as a function of age, sex, cannabis experience, and patient population.
Implementation of Electronic Cannabis Screening in UCLA Health, *Lillian Gelberg, MD*	Issue: Dr. Lillian Gelberg presented findings geared at implementing an electronic cannabis and tobacco-use screening instrument in UCLA Health system to understand prevalence of cannabis use and associated outcomes among patients. With nearly 140,000 patients screened in just 2 years, 16% endorsed current (past 3 months) use with one-third of these respondents screening positive for moderate to high risk of CUD. While over 50% of patients identified solely as nonmedical cannabis users, over half of these individuals endorsed using cannabis for symptom management, suggesting that many more people use cannabis for some kind of therapeutic purpose even when not self-identifying their use as medical. Recommendations: (1) Institute rigorous surveillance of cannabis use measuring reasons for use (medical vs. nonmedical), frequency of use, types of products used, and assessing outcomes using validating measures. (2) Link cannabis-use screeners in health care settings to EHRs to develop population-based data associating cannabis use with health outcomes.
Cannabis and Atrial Fibrillation, *Gregory Marcus, MD, MAS*	Issue: Dr. Gregory Marcus delved into his research probing the association between cannabis use and atrial fibrillation, the most common arrhythmia and a risk factor for stroke, heart failure, myocardial infarction, and dementia. Using the NIH-funded Eureka Research Platform to leverage mobile technology and data collection, studies are underway to measure the relationship between cannabis use in real time with a cardiovascular predictor of atrial fibrillation, cardiac ectopy. Recommendations: (1) Leverage mobile data technologies to identify health outcomes associated with cannabis use in real time. (2) Improve product labeling and product quality control. (3) Develop patient education materials related to risks of cannabis use, how to read labels, locations of regulated dispensaries, and improve access to evidence-based information and lower risk use guidelines.
Challenges to Conducting Medicinal Cannabis Research, *Igor Grant, MD*	Issue: Dr. Igor Grant described the challenges faced by the cannabis research community including lack of ecologically relevant cannabis and cannabis-based products, significant regulatory hurdles that severely delay progress, and lack of funding to support this research. Recommendation: (1) Institutions should engage with federal and state lawmakers and policy makers to encourage research and ease restrictions to develop a path toward establishing evidence-based information that is urgently needed to ensure public health and safety.

CUD, cannabis-use disorder; EHR, electronic health record.

## Cannabis and Public Safety

Thomas Marcotte (UCSD) led off the Cannabis and Public Safety session (Table 6) by presenting findings from a randomized clinical trial of smoked cannabis and driving simulator performance. Neal Garg (UCLA) presented information highlighting the development of a novel method for detecting THC in breath. Yuyan Shi (UCSD) discussed her work on the impacts of cannabis laws on crime and the disparity in crime. Dorie Apollonio (UCSF) concluded the session reporting on the analysis of California Poison Control System data regarding accidental ingestion of cannabis.

**Table 6.** Cannabis and Public Safety Panel

**Presentation**	**Key policy issues and recommendations**
The Impact and Detection of Driving Under the Influence of Cannabis, *Thomas Marcotte, PhD (Keynote and session moderator)*	Issue: Dr. Marcotte's presentation noted that although THC exposure was associated with worse driving performance, not all individuals showed significant driving declines. There was a disconnect between perceived impairment vs. actual performance, users ended up with similar levels of impairment regardless of the THC cigarette content, and THC blood concentrations did not correlate with driving abilities. This evidence suggests that *per se* laws, which state that drivers would be considered driving under the influence of cannabis if they have a blood THC concentration above a certain threshold, are not supportable. Recommendation: Additional methods for determining impairment due to cannabis use, and recency of use, are needed.
Marijuana Breathalyzer Technology, *Neil Garg, PhD*	Issue: Cannabis (cannabis having greater than 0.3% delta-9-THC) usage impactions reaction time, spatial judgment, and temporal perception, yet bodily fluid samples are not reliable for judging recent usage. Recommendation: Dr. Garg's presentation highlighted the need for a fair and accurate means to test for impairment from cannabis use and noted that breathalyzer technology has the potential to address questions regarding recency of use as delta-9-THC persists on the breath for about 3–4 h after consumption.
Impacts of Cannabis Laws on Crimes and Disparity in Crimes, *Yuyan Shi, PhD*	Issue: Dr. Shi's presented her research showing that while there was a reduction in arrest rates with “decriminalization,” disparities were reduced in adults but not youth. “Legalization,” on the contrary, reduced arrest rates in adults but not in youth, with no reduction in disparities in either group. Recommendations: If the sole consideration is to reduce cannabis possession arrest rates, then: Both decriminalization and legalization can achieve the goal among adults. Even after cannabis is decriminalized, legalization can still further reduce arrest rates. Decriminalization has additional benefits in that it reduces arrest rates among youths and racial disparity between blacks and whites. California should consider other strategies to reduce arrest rates among youth and reduce racial disparities.
Accidental Ingestion of Cannabis, *Dorie Apollonio, PhD, MPP*	Issue: Dr. Apollonio discussed the analysis of California Poison Control System data, showing that cannabis poisonings increased significantly after legalization, primarily driven by poisonings in children (younger than 13). These usually involved a child or caregiver mistaking a cannabis product for food, with the children often eating an entire package. Recommendations: (1) Regulations should be updated to require plain packaging, better warning labels, and child-resistant packaging. (2) Per-package levels of THC should be reduced, and individual packaging would help minimize poisonings. (3) Recommend use of medication lockboxes for all cannabis products. (4) Regulatory hurdles in studying acute cannabis use should be reduced.

## Concluding Thoughts

Overall, the California Cannabis Research Briefing was received well by the attendees, with robust discussions occurring following each section and throughout the day. In an exit survey following the event, attendees noted that they valued the breadth of topics covered and the opportunities for engagement across research expertise and policy foci. Continued engagement between state policymakers and the research community could help accelerate responses to challenges that arise from cannabis adult use legalization.

## Abbreviations Used

CUD: cannabis-use disorder
DCC: Department of Cannabis Control
EHR: electronic health record
POS: point of sale
THC: tetrahydrocannabinol

